# The effectiveness of telepsychiatry: a thematic review

**DOI:** 10.1192/bjo.2021.183

**Published:** 2021-06-18

**Authors:** Gunjan Sharma, Karrish Devan

**Affiliations:** South London and Maudsley NHS Foundation Trust

## Abstract

**Aims:**

The authors conducted a thematic review on the effectiveness of Telepsychiatry in light of the COVID-19 pandemic. The study aimed to clarify the effectiveness of Telepsychiatry, providing an evidence base for the growing use of Telepsychiatry.

**Method:**

The authors searched three databases - Cochrane, PubMed and PsychINFO - using the terms virtual consultation/telepsychiatry/video consultation AND psychiatry/mental illness.

The authors excluded all papers that were not in English and that did not focus on the psychiatric consultation.

**Result:**

961 papers were identified, reduced to 321 using exclusion criteria and removal of duplicates. Using thematic analysis the authors found five themes that occurred across all papers in relation to the effectiveness of Telepsychiatry.

Patient & Clinician Satisfaction

There is consistently high patient satisfaction with telepsychiatry but lower clinician satisfaction, often as a result of cynicism and a lack of familiarity. Clinician satisfaction increases when clinicians trial Telepsychiatry and become more positive about its uses.

Diagnostic Reliability

Telepsychiatry was found to have high levels of inter-rater reliability equivalent to face-to-face consultations for common disorders including mood and psychotic disorders, substance misuse and dementia. It was also found to have high levels of diagnostic reliability across age groups.

Outcomes

Telepsychiatry has been found to reduce symptoms of common psychiatric disorders and improve quality of life in a variety of environments including emergency departments, inpatient units and prisons. Telepsychiatry increases access to specialised services resulting in quicker access to treatment and reduction in admissions.

Technology

Without adequate internet connectivity clinicians are unable to conduct an appropriate mental state examination and the therapeutic relationship becomes challenging. Inadequate technology can impact the effectiveness of Telepsychiatry amongst those who are socioeconomically disadvantaged and may not have access to appropriate technology.

Professional Guidance

There is a concerning lack of guidance around the use of Telepsychiatry. Without clear protocols there is a lack of standardisation and clinicians are unwilling to integrate Telepsychiatry into their practice. Main concerns raised are around confidentiality, consent, the appropriateness of certain patient groups and emergencies.

**Conclusion:**

This review found evidence for the effectiveness of Telepsychiatry with greatest emphasis on technology and patient satisfaction. The main barrier is the reluctance amongst clinicians to facilitate Telepsychiatry into their practice, often due to cynicism and a lack of familiarity. The authors recommend training in the uses of Telepsychiatry and the provision of professional guidance from medical bodies to allay concerns and provide clear standards.

